# Exploring Homeostasis Restoration as a Potential Therapeutic Avenue for Addressing Obesity and Sleep Disorders

**DOI:** 10.1002/alz.088259

**Published:** 2025-01-03

**Authors:** Rakesh Bhaskar, Krishna Kumar Singh, Shampa Ghosh, Madhumita Mahapatra, Jitendra Kumar Sinha

**Affiliations:** ^1^ Yeungnam University, Gyeongsan, Gyeonsang Korea, Republic of (South); ^2^ Symbiosis Centre for Information Technology, Pune, Maharastra India; ^3^ GloNeuro Academy, Noida, Uttar Pradesh India; ^4^ Delhi Technical Campus, Greater Noida, Uttar Pradesh India

## Abstract

**Background:**

Obesity is caused by the buildup of excess body fat, which upsets homeostasis. Genetic, epigenetic, and behavioural variables all have a role in the pathophysiology of obesity. In turn, obesity throws off the sleep cycle, leading to sleep problems. Sleep is essential for preserving homeostasis, adjusting to environmental changes, and saving energy. The contemporary way of living frequently disrupts sleep cycles, which is bad for health. Many pathophysiological disorders are caused by changes in homeostasis, and obesity is one important consequence.

**Method:**

This study examines the complex connection between fat and sleep, focusing on how sleep disturbances affect energy homeostasis. The study explores the significance of sleep in evolution, as well as its anabolic properties and role in restoring glycogen stores that are used up during waking hours. It also looks at how modern lifestyles affect sleep, establishing a connection between energy balance, obesity, and sleep behaviour.

**Result:**

Reduced sleep length and poor quality of sleep are important obesity risk factors that negatively impact energy homeostasis. The review emphasises the necessity of interventions aimed at managing obesity and sleep disorders in order to enhance both the quantity and quality of sleep. People who are at a higher risk of obesity should maintain good sleep hygiene and sleep discipline.

**Conclusion:**

Comprehending the relationship between fat and sleep is crucial, given the noteworthy overlap between disorders such as sleep apnea and imbalanced energy. The review underscores the significance of addressing obesity and sleep disorders together in order to lessen related health effects. To find evidence that such therapies are effective in decreasing obesity‐related health outcomes, more study is required.

